# Overexpression of *AHL9* accelerates leaf senescence in *Arabidopsis thaliana*

**DOI:** 10.1186/s12870-022-03622-9

**Published:** 2022-05-19

**Authors:** Yusen Zhou, Xiaomin Zhang, Jing Chen, Xiaopeng Guo, Hongyan Wang, Weibo Zhen, Junli Zhang, Zhubing Hu, Xuebing Zhang, José Ramón Botella, Toshiro Ito, Siyi Guo

**Affiliations:** 1 State Key Laboratory of Crop Stress Adaptation and Improvement, School of Life Sciences, Henan University, Kaifeng, 475004 China; 2 State Key Laboratory of Cotton Biology, School of Life Sciences, Henan University, Kaifeng, 475004 China; 3 Plant Genetic Engineering Laboratory, School of Agriculture and Food Sciences, The University of Queensland, Brisbane, Queensland 4072 Australia; 4 Division of Biological Science, Nara Institute of Science and Technology, Ikoma, Nara 630-0192 Japan

**Keywords:** *Arabidopsis thaliana*, AT-hook like proteins, Ethylene, Leaf senescence, Transcription regulation

## Abstract

**Background:**

Leaf senescence, the final stage of leaf growth and development, is regulated by numerous internal factors and environmental cues. Ethylene is one of the key senescence related hormones, but the underlying molecular mechanism of ethylene-induced leaf senescence remains poorly understood.

**Results:**

In this study, we identified one AT-hook like (AHL) protein, AHL9, as a positive regulator of leaf senescence in *Arabidopsis thaliana*. Overexpression of *AHL9* significantly accelerates age-related leaf senescence and promotes dark-induced leaf chlorosis. The early senescence phenotype observed in *AHL9* overexpressing lines is inhibited by the ethylene biosynthesis inhibitor aminooxyacetic acid suggesting the involvement of ethylene in the *AHL9*-associated senescence. RNA-seq and quantitative reverse transcription PCR (qRT-PCR) data identified numerous senescence-associated genes differentially expressed in leaves of *AHL9* overexpressing transgenic plants.

**Conclusions:**

Our investigation demonstrates that *AHL9* functions in accelerating the leaf senescence process via ethylene synthesis or signalling.

**Supplementary Information:**

The online version contains supplementary material available at 10.1186/s12870-022-03622-9.

## Background

Senescence is an intricate and highly orchestrated process in the plant’s life cycle. A range of biological events occur at the physiological, biochemical, and molecular levels during this period, including chloroplast degradation, hydrolization of macromolecules, reduction of cytoplasmic volume, and decrease in cellular metabolic activity. The most noticeable feature in leaf senescence is the rapid degradation of chlorophyll during chloroplast disassembly, which leads to the yellowing of leaves [1]. During senescence, leaf nutrients are remobilized and relocated from the dying leaves to seeds or other storage tissues, thereby contributing to the fitness and survival of plant [2–4]. Although senescence is an active process to relocate nutrients from old tissues, precocious senescence will shorten the growth stage of crops and result in reduced yield and crop quality [2, 5, 6].

Senescence involves massive transcriptional changes, and a large number of senescence-associated genes (*SAGs*) have been identified. The expression of *SAGs* is regulated by senescence-related transcription factors TFs such as NACs (NAM [No Apical Meristem], ATAF2 [*Arabidopsis* Transcription Activation Factor2] and CUC2 [Cup-shaped Cotyledon2]), MYBs, bZIPs (basic region/leucine zipper motifs) and WRKYs TFs [7–10]. Incubation of detached leaves in the dark is highly effective for induction of *SAGs*, leaf yellowing and chlorophyll loss. Therefore, it has been widely used as a model system for the study of leaf senescence [11].

Although leaf senescence occurs in an age-dependent manner, the process is also greatly affected by multiple endogenous and environmental signals coordinating the life span of leaves to optimize plant fitness. Endogenous signals such as plant hormones play vital roles in the senescence process via complex interconnecting pathways. Positive regulators include abscisic acid, ethylene, jasmonic acid, salicylic acid, brassinosteroid and strigolactone, in contrast, cytokinin, gibberellic acid and auxin suppress senescence [12, 13]. Ethylene has long been established as a key hormone regulating the timing and progression rate of leaf senescence [14, 15]. In vitro application of ethylene induces premature leaf senescence, while application of inhibitors of ethylene biosynthesis or action can delay leaf senescence symptoms [16, 17].

Transcriptional analysis has shown that the expression levels of a number of genes encoding ethylene biosynthesis, such as 1-aminocyclopropane-1-carboxylic acid synthase (ACS) and ACC oxidase (ACO), and signaling components increase in senescence leaves [16], indicating ethylene signaling is involved in the regulation of senescence leaves. This is further supported by the extended leaf longevity of ethylene-insensitive mutants that are defective in ethylene signaling transduction, such as *etr1* (*ethylene resistant 1*), *ein2* (*ethylene insensitive 2*) and *ein3* [18–20]. To date, the regulation of ethylene seems to be achieved by the EIN2-EIN3-miR164-NAC2 signalling cascade. EIN2 regulates *miR164* expression, as well as its downstream target gene *ORESARA1 (ORE1)* *ORE1*/*NAC2* [21]. EIN3 acts at the downstream of EIN2 to promote chlorophyll degradation by affecting chlorophyll catabolic genes [22]. Although the ethylene biosynthetic pathway and downstream key elements involved in ethylene signal transduction have been extensively studied through genetic approaches, the transcriptional network leading to leaf senescence remains largely unknown.

AT-HOOK MOTIF CONTAINING NUCLEAR LOCALIZED (AHL) proteins are transcription factors featured with two conserved structural units: a plant and prokaryote conserved (PPC) domain, involved in protein–protein interactions, and one or two DNA-binding AT-hook motif(s) [23–27]. The AT-hook motif contains a conserved palindromic core sequence, Arg-Gly-Arg, capable to bind to the minor groove of AT-rich B-form chromosomal DNA, thus changing its architecture and controlling the expression of corresponding genes [25, 28–30]. AHL family proteins have been proposed to regulate plant growth and development. AHL22 acts as a chromatin remodeling factor that regulates *FT (FLOWERING LOCUS T*) expression to promote flowering [31]. Several AHLs are involved in hormonal homeostasis and response, especially gibberellins, cytokinins and jasmonic acid [28, 32, 33]. *ESC*/*AHL27* (*ESCAROLA*) and *SOB3*/*AHL29* (*SUPPRESSOR OF PHYTOCHROME B-4 #3*) act redundantly to repress hypocotyl elongation in response to light [24, 34]. Overexpression of *AHL27* in *Arabidopsis* delays senescence and increases post-harvest storage life, but the molecular mechanism is unclear [29]. Recently, it has been reported that *SOB3*/*AHL29* repress petiole growth by antagonizing PIF-mediated transcriptional activation of genes associated with growth and hormonal pathways [35].

Although the fact that one member of the AHL clade A is involved in leaf senescence, the detailed mechanism by which AHL transcription factors regulate leaf senescence still remain largely unknown. Here, we identified AHL9 as a positive regulator of leaf senescence in *Arabidopsis*. The overexpression of *AHL9* causes a severely early senescence phenotype. Moreover, we showed in the current study that *AHL9* not only promotes dark-induced but also ethylene-induced leaf chlorosis. Our RNA-seq analysis showed that the expression of multiple *SAGs* is altered in *AHL9* overexpressing lines. Our results provide new insights into the molecular mechanism of leaf senescence highlighting *AHL9* as a prominent regulatory component in dark-induced and ethylene-induced leaf senescence.

## Results

### Overexpression of *AHL9* results in premature leaf senescence

**Fig. 1:**
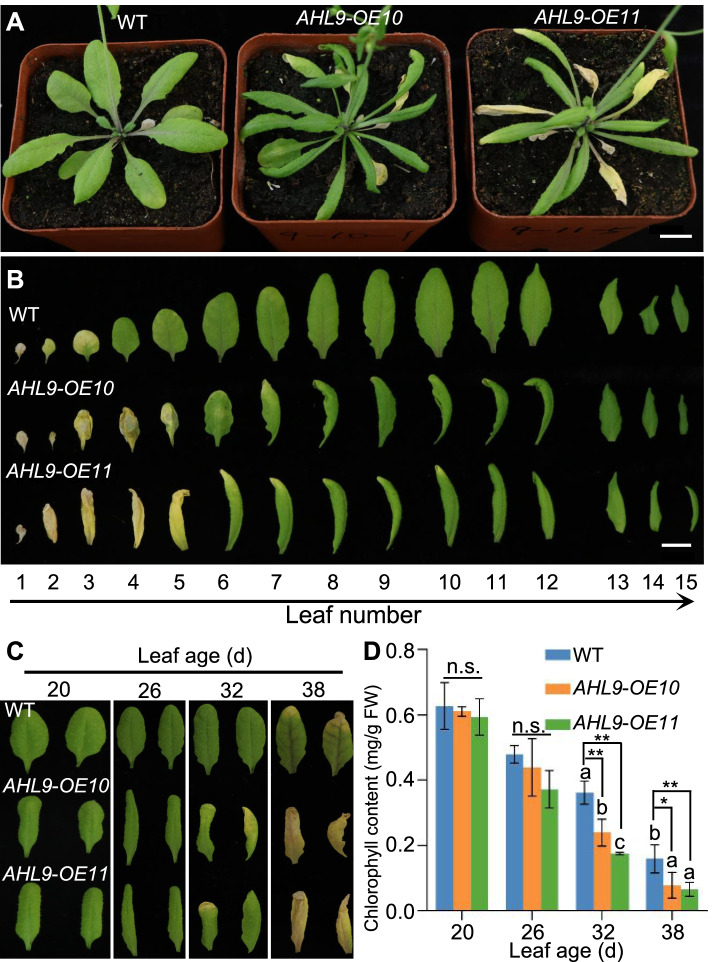
Overexpression of *AHL9* causes early senescence in rosette leaves. **A** Leaf phenotypes of 32-d-old WT, *AHL9*-*OE10* and *AHL9*-*OE11* overexpressing plants. Scale bar = 1 cm. **B** Leaves detached from (A) and arranged according to their age. Rosette leaves were numbered from bottom to top with the first leaf being the oldest. Scale bar = 1 cm. **C** Representative images of the fourth and fifth rosette leaves detached from WT, *AHL9*-*OE10* and *AHL9*-*OE11* plants of the different ages. **D** Chlorophyll contents of the leaves shown in (C). *P < 0.05, **P < 0.01, Data indicate means ± SD, n = 3. Statistical analyses were performed using one-way ANOVA. Means with different letters above the bars indicate statistically significant results (*P* < 0.05). The experiment was conducted three times with similar results

AHL family proteins include clade A and clade B subfamilies [24, 26]. To further explore the functions of AHL clade B subfamily, a phylogenetic tree was generated and analysed (Fig. S1). *AHL9* was chosen to study its biological functions. Bioinformatics analysis demonstrates that *AHL9* genome fragments contain 5 exons and 4 introns, the AHL9 protein has two AT-hook motifs and one DUF296 (plant and prokaryote conserved (PPC)/domain of unknown function #296 domain) (Fig. S2). AHL11 is the closest homolog of AHL9 in *Arabidopsis* genome, which exhibits 62.2% sequence identity with AHL9 based on full-length alignment (Fig. S3). Through the quantitative reverse transcription PCR (qRT-PCR) assay, various expression abundancy of *AHL9* was detected**.** However, a higher expression in the aging tissues (the fourth and fifth rosette leaves) than in proliferative tissue (the siliques) was observed, indicating *AHL9* may have the role during the senescence process (Fig. S4). To test the biological function of *AHL9*, *35S::AHL9* transgenic *Arabidopsis* lines were generated. Two independent transgenic lines (*35S::AHL9*-*OE10* and *OE11*) were further analysed. Compared with WT, both transgenic lines displayed premature leaf senescence at 32 d (Fig. 1A). In addition to precocious leaf senescence, the rosette leaves of both *AHL9* transgenic lines became elongated and narrow (Fig. 1A). To gain a better view of the function of *AHL9* in leaf senescence, the rosette leaves of transgenic lines were compared with their corresponding WT at the same stage. As shown in Fig. 1B, when the first five leaves of *AHL9*-*OE10* and *AHL9*-*OE11* had already turned yellow, only the first three leaves became yellow in WT. In contrast with an apparent premature senescence of *AHL9* transgenic lines, the *AHL9* T-DNA insertion mutant was almost like the WT (Fig. S5). Phylogenetic analysis showed that AHL9 hold the similarity with AHL11, AHL5 and AHL12, indicating the possible functional redundancy. As AHL11 exhibited the highest similarity with AHL9, we generated the double mutants that lack of both AHL9 and AHL11. All plants (WT, *ahl11*, *ahl9 ahl11-1* and *ahl9 ahl11-2*) exhibited the similar rate in leaf senescence, implying the higher-order redundancy among the proteins belonging to AHLs family. (Fig. S6).

**Fig. 2:**
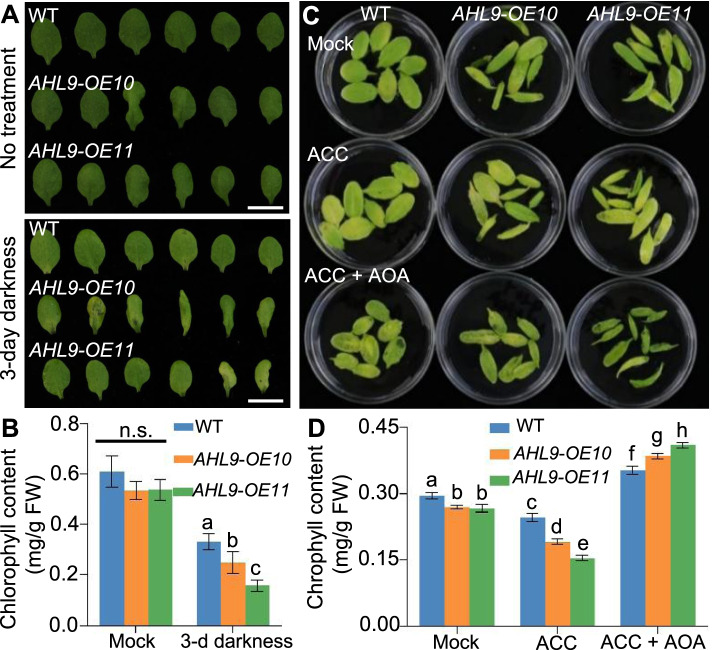
*AHL9* is involved in dark-induced and ethylene-induced leaf senescence. **A** Phenotype of detached leaves from 3-week-old WT and two independent *AHL9* overexpressing lines subjected to dark treatment. Detached leaves were incubated in MES buffer for 3 d under dark conditions. Scale bar = 1 cm. **B** Chlorophyll content of detached leaves from (A). The data were analyzed using one-way ANOVA analysis. Means with different letters above the bars indicate statistically significant results (*P* < 0.05). Data indicate means ± SD, *n* = 3. The experiment was conducted three times with similar results. **C** Phenotype of detached leaves from 3-week-old WT and two independent *AHL9* overexpressing lines ACC or ACC + AOA in the dark. Detached leaves were treated with MES buffer, 100 µM ACC or 100 µM ACC + 500 µM AOA for 3 d under the dark conditions. **D** Chlorophyll contents in leaves from (C). The data were analyzed using one-way ANOVA analysis. Means with different letters above the bars indicate statistically significant results (*P* < 0.05). Data indicate means ± SD, *n* = 3. The experiment was conducted three times with similar results

**Fig. 3:**
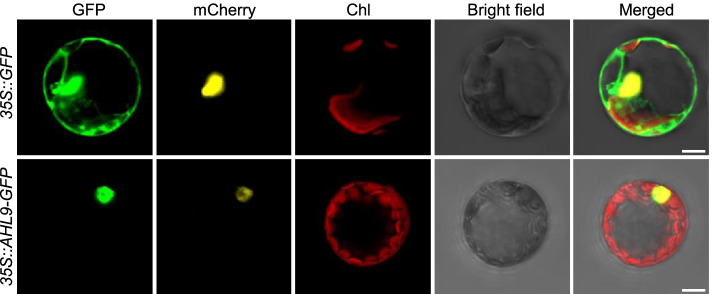
AHL9 is localized exclusively in the nucleus. *Arabidopsis* protoplasts were co-transfected with expression cassettes containing either *35S::GFP* and *35S::H2B-mCherry* or *35S::AHL9-GFP* and *35S::H2B-mCherry*. GFP signals were detected using a laser confocal scanning microscopy. H2B-mCherry was used as a nuclear marker. From left to right are green fluorescence signal, nuclear marker, chlorophyll red auto fluorescence, bright-field and merged images, respectively. Scale bars = 10 μm

**Fig. 4:**
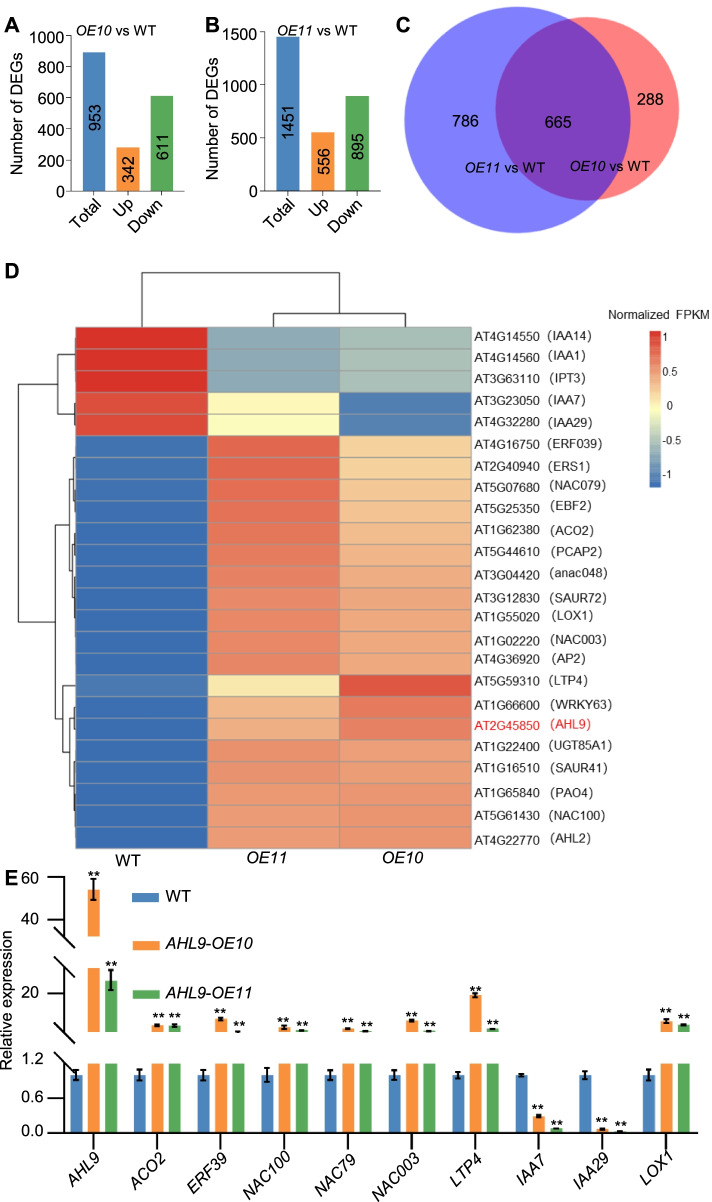
RNA-seq analysis of WT, *AHL9*-*OE10* and *AHL9*-*OE11* transgenic lines. **A-C** Diagram showing differentially expressed genes (DEGs). DEGs were classified according to their expression fold-changes (FC) in the pairwise genotypic comparison between *AHL9*-*OE10* or *AHL9*-*OE11* and WT leaves (q-value < 0.01, |log_2_(fold change)|> 1). **D** Heatmap of senescence-associated genes (SAGs) in DEGs of *AHL9*-*OE10*, *AHL9*-*OE11* and WT. The color bar indicates the normalized gene expression. **E** Expression levels of ten randomly chosen DEGs identified in the RNA-seq experiments. qRT-PCR was performed using cDNA from 30-d old leaves of *AHL9*-*OE10*, *AHL9*-*OE11* and WT. qRT-PCR values are expressed as the mean ± SD compared to that of the internal control (*UBQ10*). The fold change of the qRT-PCR was determined by the efficiency method (2^−ΔΔCT^). Error bars indicate SD. n = 3, *t*-test, **P < 0.01. Assays were done in triplicate

**Fig. 5:**
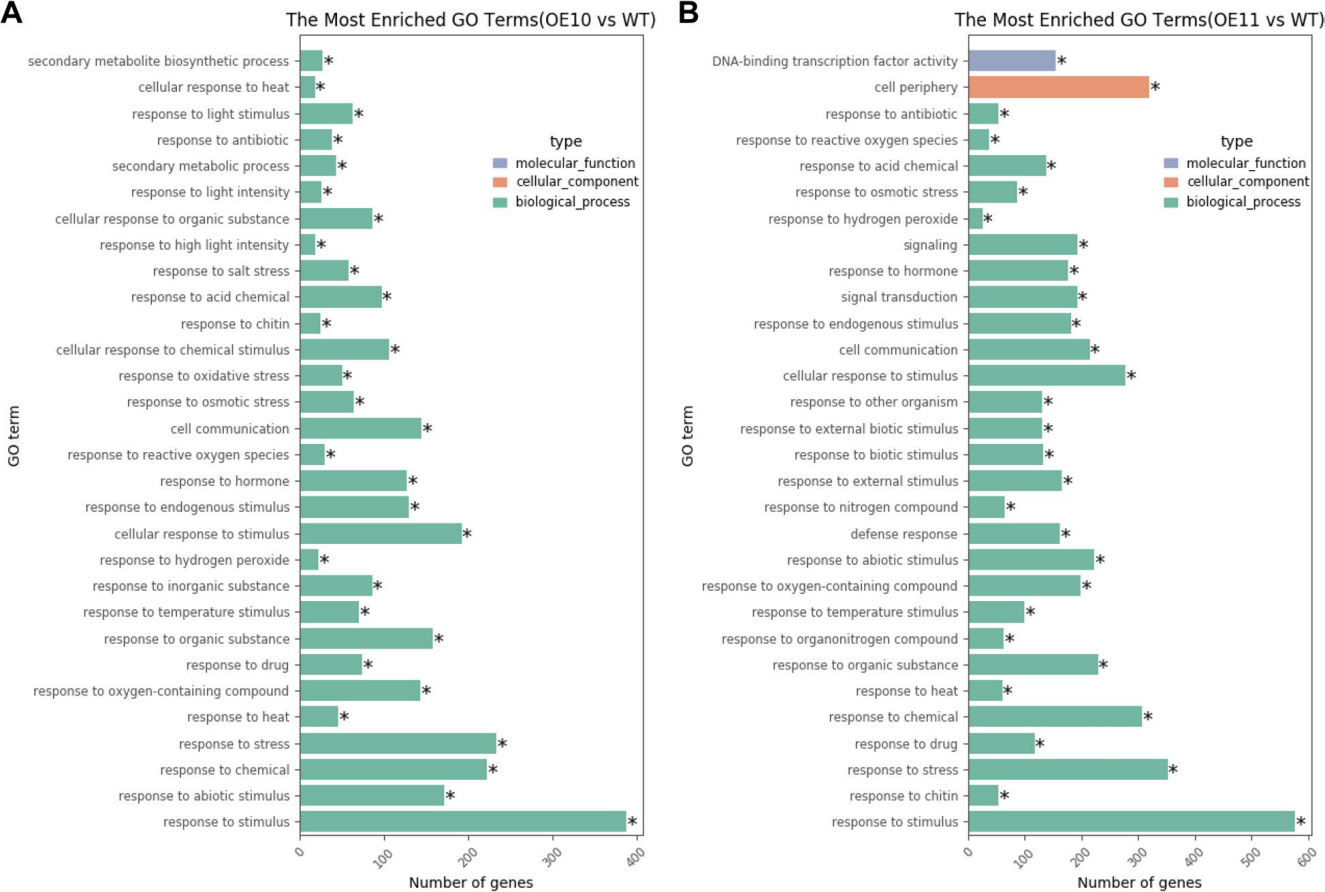
Gene ontology analysis of DEGs. Gene ontology terms of DEGs identified in the RNA-seq experiments. **A-B** *AHL9*-*OE10* vs WT (**A**), and (**B**) *AHL9*-*OE11* vs WT. CC, cellular component. MF, molecular function. BP, biological process

To further analyse the development of age-related senescence in WT and *AHL9* overexpressing plants, the fourth and fifth rosette leaves detached from WT and *35S::AHL9* transgenic lines were used for further analysis in the different stages. At 20 and 26 d after germination, there was no significant difference in leaf colour between transgenic lines and WT. However, at 32 d after sowing, the fourth and fifth rosette leaves of *35S::AHL9* transgenic lines became yellow, while the same stage leaves of WT were still green. The yellowing of these leaves progressed rapidly in the *AHL9-OEs* transgenic lines, and the majority of them become completely yellow at 38 d after germination, at this stage, WT plants only started to show signs of leaf senescence with only the tip of the leaf turning yellow (Fig. 1C). This is consistent with previous reports showing that senescence symptoms usually start from the tip and outer edge of a rosette leaf at a given age [36]. Chlorophyll quantification assays confirmed the visual phenotypic observations showing significantly lower chlorophyll content in the fourth and fifth rosette leaves in *AHL9* overexpressing transgenic lines in 32 d and 38 d-old plants, compared with WT plants (Fig. 1D).

### AHL9 regulates dark- and ethylene-induced leaf senescence

Incubation of detached leaves in darkness is often used as an effective method to stimulate synchronous senescence [37]. To further probe the potential roles of *AHL9* in leaf senescence, we examined dark-induced leaf senescence in three-week-old WT and *35S::AHL9* transgenic plants. Rosette leaves of the same age detached from 3-week-old WT and *35S::AHL9* plants were similar in colour before dark treatment; however, after 3 days darkness, *35S::AHL9* leaves exhibited stronger leaf chlorosis than WT (Fig. 2A). The phenotypic observations were confirmed by chlorophyll assays showing a more pronounced decrease in chlorophyll levels in the *AHL9-OE* leaves compared to WT (Fig. 2B), suggesting that dark-induced leaf senescence is promoted by the overexpression of *AHL9*.

Phytohormones play critical roles in leaf senescence, therefore we queried whether the overexpression of *AHL9* altered the ethylene- and ABA-induced senescence. For this purpose, we first examined the darkness-induced senescence of detached leaves upon treatment with either the ethylene precursor 1-aminocyclopropane-1-carboxylic acid or ACC plus aminooxyacetic acid (AOA), an ethylene biosynthesis inhibitor, respectively. As expected, pre-treatment with ACC increased senescence in WT and *35S::AHL9* leaves (Fig. 2C, D). In contrast, the enhanced senescence observed in *35S::AHL9* leaves was suppressed by treatment with ACC and AOA (Fig. 2C, D), suggesting that the role of *AHL9* in senescence is dependent on ethylene. Unlike ethylene, ABA treatment did not significantly increase the difference of chlorophyll content between WT and *AHL9* overexpression transgenic lines (Fig. S7). These results indicate the involvement of *AHL9* in ethylene-induced leaf senescence.

### AHL9 is a nuclear localized AT-hook protein

To establish the AHL9 subcellular localization, an AHL9-GFP fusion construct or GFP (control) were transiently expressed in *Arabidopsis* mesophyll protoplasts under the control of the cauliflower mosaic virus *35S* promoter. The nuclear marker protein H2B-mCherry was co-expressed to visualize nuclei [38]. In the GFP controls, green fluorescence was observed throughout the protoplasts (Fig. 3). Green fluorescence in AHL9-GFP transfected protoplasts was restricted to the nuclei and overlaped with the yellow fluorescence of the nuclear marker, indicating that AHL9-GFP is located in the nucleus (Fig. 3).

### Identification of differentially expressed genes in *AHL9* overexpression lines

In order to investigate the possible roles of *AHL9* in leaf senescence, we performed genome-wide expression profiling of *AHL9-OEs* and WT plants under normal growth conditions. Leaves from 30-d old WT and both *AHL9-OEs* transgenic lines were used for RNA-seq experiments. In total 20 million uniquely mapped reads per sample with high reproducibility among all three biological replicates were generated (Table S1). Analysis of *AHL9-OE10* vs WT identified 953 genes with statistically significant differences in gene expression (log_2_^(fold_change)^ > 1, q-values < 0.01), including 342 up-regulated and 611 down-regulated genes (Fig. 4A-B and Table S2). In the case of *AHL9*-*OE11*, 1451 genes showed statistically significant differences with WT, with 556 up-regulated and 895 down-regulated genes. When both datasets are put together, a common set of 665 differentially expressed genes (DEGs) were identified in *AHL9*-*OE10* and *AHL9*-*OE11* vs WT (Fig. 4C). Interestingly, some of the genes with altered expression belong to NAC, WRKY, IAA, and AP2 transcription factors, ACC oxidase, and the ETHYLENE RESPONSE SENSOR (Fig. 4D). Principal component analysis (PCA) and correlation analysis of all datasets indicated strong RNA-seq reliability (Fig. S8-S9). qRT-PCR analysis of 10 randomly chosen DEGs are consistent with the RNA-seq results (Fig. 4E, Table S3). Gene Ontology analysis highlighted the expression changes of genes involved in response to stimuli, hormone response, biological process and biological regulation (Fig. 5). Given that gene regulatory networks composed of interactions between TFs (transcription factors) and their targets have been implicated in controlling leaf senescence, we conducted GO enrichment analysis for these TFs. Indeed, we found most of these TFs were significantly enriched in GO terms that may associate with biological process, such as the RNA biosynthetic or metabolic process, cellular macromolecule biosynthetic process and nitrogen compound metabolic process (Fig. S10). These results provided molecular evidence supporting premature leaf senescence in *AHL9* overexpression lines.

## Discussion

In recent years, our knowledge about the molecular mechanisms triggering leaf senescence has expanded significantly. Transcriptomic analysis of leaf senescence revealed the expression changes of thousands of *SAGs*, however, only a small portion of them have been functionally characterized [8]. Besides, factors on the top of the regulation module regulating diverse *SAGs* and/or other functional genes have also been identified through either loss-of-function and/or gain-of-function studies in model plants such as *Arabidopsis* and rice [2, 23]. Among these regulators, transcription factors are interesting candidates as they can influence the expression of multiple genes during the senescence process.

The study of *AHL* family genes revealed different roles in plant growth and development, such as hypocotyl elongation, flower development, gibberellin biosynthesis and leaf longevity [25, 28, 31, 34]. Although knowledge about the functional role of AT-hook motif proteins is still very limited in plants. Notably, Ectopic *ORE7/ESC* delays leaf senescence, which probably up-regulate genes that suppress senescence and down-regulate genes that enable the progression of the senescence process through modification of chromatin architecture[29]. In this study, we identify and characterize an AHL protein, AHL9, involved in leaf senescence. Overexpression of *AHL9* results in early senescence in *Arabidopsis* (Fig. 1). Transgenic plants overexpressing *AHL9* also exhibited accelerated dark-induced leaf senescence (Fig. 2A). In contrast, the lack of senescence-related phenotype in the *ahl9* mutant may be due to the existence of functionally redundant genes, since 4 paralogs were identified in *Arabidopsis*, including AHL5, AHL12, AHL9, and AHL11 [26]. The lack of phenotype in the *ahl9* mutant could also be explained by functional compensation from other senescence-associated pathways, since leaf senescence is the integrated result of various pathways that incorporate numerous endogenous factors and environmental signals [8, 23]. Taken together, these results suggest that AHL9 behaves like an early senescence-activator that influences both age and dark-induced leaf senescence.

Although ethylene has been known for many decades to be a senescence-inducing plant hormone, the molecular mechanism underlying ethylene-mediated senescence remains largely unknown. Comparative transcriptome analyses have revealed a number of ethylene biosynthesis and signalling genes, with elevated transcript levels in senescing leaves, supporting the idea that sensitivity of a leaf to ethylene might account for the age-dependent leaf senescence [16, 18]. Here, we show that AHL9 is involved in the control of leaf senescence through ethylene synthesis or signalling since pre-treatment with the ethylene biosynthesis inhibitor AOA, can effectively repress the increased senescence observed in *AHL9* overexpressing transgenic lines. Consistent with this data, some ethylene synthesis and signalling genes are induced in the RNA-seq data, although the genes with altered expression may not be the direct targets of AHL9 (Fig. 4D). Intriguingly, we also find some of these TFs that show DEGs are significantly enriched in GO terms that may associated with stress response such as response to abiotic stimulus, ethylene-activated signaling pathway and hormone-mediated signaling pathway. Ethylene is sensed by a five-member family of ethylene receptors on the endoplasmic reticulum [39], then this binding inactivates a Raf-like Ser/Thr kinase, CTR1 (CONSTITUTIVE TRIPLE RESPONSE1), thereby releases the C-terminal end of the positive regulator, EIN2, to the nucleus and stabilizes EIN3 and ETHYLENE INSENSITIVE3-LIKE1 (EIL1), which in turn, activate the expression of ethylene target genes to promote premature senescence in leaves [40–43]. Identifying specific target genes and interaction proteins of AHL9 in vivo will be the next step required to gain a more in-depth knowledge of its role in regulating leaf senescence.

## Conclusions

The current study demonstrates that one AT-hook protein, AHL9, may regulate leaf senescence via the fine-tuning of ethylene biosynthesis or signalling.

## Methods

### Plant materials and growth conditions

The *Arabidopsis thaliana* ecotype Columbia (Col-0) was used in this study [44]. The transfer DNA (T-DNA) insertional mutant *ahl9* (GK_735D06) was obtained from the Nottingham *Arabidopsis* Stock Centre (NASC). Seeds were surface sterilized in 10% (v/v) sodium hypochlorite for 10 min, washed 3 times with sterilized water, and then grown on Murashige and Skoog medium plus 3% sucrose and 0.6% agar (pH 5.8) after 2 d vernalization in darkness at 4℃. The 7-d-old seedlings were transferred into soil and were grown at 22℃ in a 16-h-light/8-h-dark cycle for additional experiments and seed production. Yusen Zhou and Jing Chen undertook the formal identification of the plant materials (T-DNA mutants, CRISPR-Cas9 mutants and transgenic plants) used in this study.

### Plasmid construction and plant transformation

*AHL9* cDNAs were obtained by RT-PCR of RNA isolated from Col-0 seedlings, the full length of *AHL9* CDS was amplified by PCR method using primers. According to the instructions of the invitrogen gateway kit (kit No.11789 (BP Clonase); No.117910 (LR Clonase)), the PCR products were cloned into pDNOR221 vector using the Gateway™ BP Clonase™ II Enzyme mix. Subsequently, *AHL9* CDS was sub-cloned into the pDEST Gateway binary vector pGWB405 between the *35S* promoter and *GFP* gene to produce the 35S::*AHL9-GFP* construct. The constructed plasmids were introduced into *Agrobacterium tumefaciens* strain GV3101 and transformed into plants using the floral dip method [45]. Transgenic plants in the T_2_ generation with T-DNA insertion at a single locus were selected by kanamycin resistance, and T_3_ homozygotes were used for all analyses. These primers were listed in Table S3.

### Generation of *ahl11* and *ahl9 ahl11* CRISPR-Cas9 mutants

For *AHL11* CRISPR-Cas9 mutant generation, first, the sgRNAs of *AHL11* were designed using the CRISPOR online internet (http://crispor.tefor.net/), we selected two sgRNAs (T1sgRNA: GAGGAGGAGGACCAGAACCG, T2sgRNA: ACAGACGTGTCACCTGAAGG). Second, two sgRNAs were constructed into pCAMBIA1300-pYAO-cas9 vector [46]. Finally, the constructed vectors were introduced into WT and *ahl9* mutants using the *Agrobacterium* mediated floral dip method respectively. The stable inheritance CRISPR-Cas9 mutants were confirmed through PCR and Sanger sequencing.

### Dark-induced senescence assay

For the dark-induced senescence assay, the fourth and fifth rosette leaves were carefully detached from 3-week-old soil-grown *Arabidopsis*. Detached rosette leaves were incubated on MES buffer (0.5 × MS, 3 mM MES, pH 5.8) in complete darkness for 3 d and sampled for analyzing leaf senescence and chlorophyll content.

### Hormone induced leaf senescence assay

The fourth and fifth leaf of 3-week-old plants were detached and floated on 3 mL of MES buffer (0.5 × MS, 3 mM MES, pH 5.8) supplemented with or without ABA (10 μM, 50 μM, 100 μM, and 200 μM), 1-aminocyclopropane-1-carboxylic acid (ACC, 100 μM) and 100 μM ACC plus ethylene biosynthesis inhibitor (AOA, 500 μM). The petri dishes were sealed with parafilm tape and wrapped with double-layer aluminum foil, then the petri dishes were put in a black box to avoid light. All hormone treatments were performed at 22℃ under the dark conditions. Three biological replicates were performed.

### Determination of chlorophyll content

Chlorophyll was measured according to the method described by Li et al. [47]. Briefly, *Arabidopsis* seedlings were weighed (W), placed into Eppendorf tubes with acetone (95%, 1 mL, V) and kept overnight under dark conditions. Samples were then centrifuged at 13,000 g for 10 min and absorbance values at 665 nm and 649 nm were obtained from the supernatant. Chlorophyll content (including chlorophyll a and b) was calculated according to the following formula: chlorophyll a = 13.95A665 – 6.88A649; chlorophyll b = 24.96A645 – 7.32A665; and total chlorophyll = (chlorophyll a + chlorophyll b) × V/W. At least three independent samples were examined, all of which produced the typical results reported in this article.

### Subcellular localization

The *35S::AHL9-GFP* and *35S::GFP* plasmids were transformed into *Arabidopsis* mesophyll protoplasts as described previously [48]. Transformed protoplasts were observed using a fluorescence microscope (Ziess confocal LSM710). H2B-mCherry were used as controls for nuclear localization [38].

### qRT-PCR analysis

Total RNA was isolated from the seedlings with Trizol reagent and DNA was digested by RNase-free DNase I. Two µg of total RNA was used for reverse transcription with M-MLV reverse transcriptase according to the supplier’s instructions (Promega). qRT-PCR analyses were performed with Roche Light Cycler 480 real-time PCR system using the SYBR Green Master Mix (Vazyme Biotech Co., Ltd.) and specific primers for PCR amplification. *UBQ10* was used as an internal control for data normalization. These primers were listed in Table S3.

### RNA-seq analysis

The sixth, seventh and eighth rosette leaves were individually collected from 30-d-old WT, *AHL9-OE10* and *AHL9-OE11*, pooled, and frozen in liquid nitrogen. The samples were stored at − 80℃ prior to RNA extraction. The RNA-seq analysis was performed at the Berry Genomics Corporation (Beijing) with three biological replicates. Briefly, The cDNA library were constructed and sequenced on HiSeq 2000 sequencing system. The clean data was produced by discarding the paired reads that one read’s number of N base is more than 5 or has more than 30% bases with a low quality value below 15. The clean reads were aligned to the reference genome of Vigna angularis using Hisat2 [49]. Then samtools and HTSeq-count were used to count the reads number of each gene and gene’s expression level was normallized as Fragments Per Kilobase Million (FPKM) [50]. DESeq2 was used to analyze differential gene expression, based on the negative binomial distribution, of the replicate samples between the treatment group and control group [51]. A threshold value of *p*-adjusted value (qvalue) < 0.01 (|log_2_foldchange|> 1) was used to obtain differentially expressed genes (DEGs). The gene ontology (GO) enrichment, with *p*-adjusted value cut-off 0.05, were performed using custom R scripts based on Bioconductor packages goseq and GO.db in order to classify DEGs into terms.

### Phylogenetic tree

Phylogenetic tree was generated using MEGA-X software. The AHL clade B family from *Arabidopsis thaliana*, some orthologs of rice and maize protein sequences were downloaded from NCBI.

### Statistical analysis

All experiments were repeated with at least three times. The presented data were expressed as the means ± SD. Statistical analyses were performed using one-way ANOVA or Student’s *t*-test.

### Accession numbers

The *Arabidopsis* Genome Initiative identifiers for the genes described in this article are as follows: AHL9 (At2g45850), AHL11 (At3g61310), LOX1 (AT1G55020), IAA1 (AT4G14560), UBQ10 (AT4G05320), ACO2 (AT1G62380), NAC079 (AT5G07680), anac048 (AT3G04420), ERF039 (AT4G16750), PCAP2 (AT5G44610), SAUR72 (AT3G12830), LTP4 (AT5G59310), UGT85A1 (AT1G22400), PAO4 (AT1G65840), WRKY63 (AT1G66600), EBF2 (AT5G25350), NAC003 (AT1G02220), NAC100 (AT5G61430), AP2 (AT4G36920), ERS1 (AT2G40940), AHL2 (AT4G22770), SAUR41 (AT1G16510), IAA7 (AT3G23050), IAA29 (AT4G32280), IAA14 (AT4G14550), IPT3 (AT3G63110).

## Supplementary information

**Additional file 1** **Fig. S1 **Phylogenic analysis of AHLs clade B family proteins in *Arabidopsis thanalia* and orthologs in *Oryza sativa L.* and *Zea mays L.*. The protein sequences were downloaded from NCBI database (https://www.ncbi.nlm.nih.gov/). And then the phylogenetic tree was generated using MEGA-X software. The numbers of the branches are the bootstrap values from 1,000 replicates. **Fig. S2 **Schematic diagrams of *AHL9* genomic and protein structure. **A** Genomic structures of *AHL9*. Arrows represent start codon. Black boxes represent exons. Crease lines represent introns. White boxes represent 5’ and 3’ untranslation regions. Scale bar, 100 bp. **B** Protein structures of AHL9. Red boxes represent AT-hook motif. Gray boxes represent DUF296 domain. **Fig. S3 **Protein sequences alignment of AHL9 and AHL11. The AHL9 and AHL11 sequences were downloaded from NCBI database (https://www.ncbi.nlm.nih.gov/). The fasta format was generated using CLUSTALW software, and then the alignment was produced with GENEDOC software. **Fig. S4 **qRT-PCR analysis of *AHL9* transcript levels in roots, leaves, stems, inflorescences, and siliques in six-week-old plants. The roots, leaves, stems, inflorescences, and siliques were sampled from three individual plants at different development stages, respectively. The fourth and fifth rosettes were used to determine the gene expression in the leaves. *Actin* was used for normalization purposes. Data indicate mean ± SD (*n* = 3). Three biological repeats were performed. **Fig. S5** Phenotypic observation of  WT and *ahl9* plants. **A** Schematic structure of *AHL9* and the positions of the T-DNA  insertion in *ahl9*. Black boxes indicate exons, lines indicate introns, empty boxes indicate upstream and downstream regions of *AHL9*. Triangles represent  the T-DNA insertion. **B** Amplification of *AHL9* in genomic DNA from the WT and *ahl9*. LP and RP primers were used for *AHL9* genomic sequence amplification. LB and RP primers were used for specific amplification of the T-DNA  insertion. **C** qRT-PCR detection of *AHL9* transcript in 28-d-old WT and *ahl9*. *UBQ10* was as an internal control. Three biological repeats were done. **P ˂ 0.01, Student's *t*-test. **D**-**E** Phenotypic observation of 28-d (**D**) and 36-d (**E**) old WT and *ahl9* plants. Scale bars, 1 cm. **Fig. S6 **Generation and phenotypic analysis of *ahl11* and *ahl9 ahl11* double mutants. **A** Schematic genomic structure of *AHL11* and the sgRNA target sites. Black boxes indicate exons, lines indicate introns, empty boxes indicate 5’UTR and 3’UTR of *AHL11*. UTR, untranslated region. **B** Sanger sequencing of the *ahl11*, *ahl9 ahl11-1* and *ahl9 ahl11-2* mutants in WT and *ahl9* backgrounds. **C** Amino acids of *AHL11*, *ahl11*, *ahl11-1*, and *ahl11-2*. **D** Phenotypic analysis of WT, *ahl9*, *ahl11*,* ahl9ahl11-1* and *ahl9ahl11-2* plants grown for 15-d, 21-d, 28-d and 34-d. Scale bars = 2 cm. **Fig. S7** The senescence phenotypes of detached leaves from *AHL9* overexpressing plants treated with ABA. Detached leaves were treated with MES buffer (Mock), 10 μM, 50 μ M or 100 μM and 200 μM ABA for 3 d under dark conditions. **Fig. S8** PCA analysis of RNA-seq of* AHL9*-*OE10 *vs WT,* AHL9*-*OE11* vs WT. PCA results for the transcript-level (FPKM) dataset of all samples. The sample with same color means samples from the same group. **Fig. S9 **Correlation analysis of the samples from the same group. R^2^ represents correlation coefficient. **Fig. S10** Gene ontology analysis of different expression transcription factors.

**Additional file 2** **Table S1 **RNA-seq maped reads of WT, *AHL9*-OE10, and *AHL9*-OE11 leaf samples.

**Additional file 3** **Table S2** Differentially expressed genes of WT, *AHL9*-OE10, and *AHL9*-OE11.

**Additional file 4** **Table S3** Primers used in this study.

## Data Availability

The RNA-seq data have been deposited into sequence read archive (SRA) database under the Bioproject PRJNA780875. The BioProject's metadata is available at https://dataview.ncbi.nlm.nih.gov/object/PRJNA780875?reviewer=sjfjtgdgtd4alusr7nf9l5has3 in read-only format. All relevant data and plant materials are available from the corresponding authors upon request.

## Abbreviations

AHL: At-Hook motif containing nuclear Localized
SAG: Senescence-Associated Gene
CUC: Cup-shaped Cotyledon
NAC: Nam, Ataf2 and Cuc2
ACC: 1-Aminocyclopropane-1-Carboxylic acid
ACS: ACC synthase
ACO: ACC oxidase
AOA: ACC plus aminooxyacetic acid
ETR1: Ethylene Resistant 1
EIN2: Ethylene Insensitive 2
PPC: Plant and Prokaryote Conserved
DUF296: Domain of Unknown Function #296 domain
DEGs: Differentially Expressed Genes
PCA: Principal Component Analysis
TF: Transcription Factor
EIL1: Ethylene Insensitive3-Like1
GO: The Gene Ontology
