# Supplementary material for: Overexpression of AHL9 accelerates leaf senescence in Arabidopsis thaliana
Source: BMC Plant Biol. 2022 May 19;22:248. doi: 10.1186/s12870-022-03622-9 (PMC9118680; doi:10.1186/s12870-022-03622-9)
Supplement: Supplementary file 4 — Additional file 4 Table S3 Primers used in this study. [file 12870_2022_3622_MOESM4_ESM.docx]

**Table S3. Primers used in this study.**

| Experiment | Primer | Sequence (5’-3’) |
| --- | --- | --- |
| Real-time PCR | UBQ10-qRT-F | GTAACATTGTGCTCAGTGGTGGTA |
|  | UBQ10-qRT-R | GATAGAACCACCAATCCAGACAC |
|  | AHL9-qRT-F1 | CATCTCCCGGAGCCATTAAATA |
|  | AHL9-qRT-R1 | TGAACAGGACTTGCAGCTATTA |
|  | AHL9-qRT-F2 | TAATAGCTGCAAGTCCTGTTCA |
|  | AHL9-qRT-R2 | TCTTCTTCTCGTTTCTTGCTCT |
|  | ACO2-qRT-F | CAATGACATGCTCAAGTCCAAA |
|  | ACO2-qRT-R | AAATCCTCAGCAAGATTCTCCA |
|  | ERF039-qRT-F | TCAGAATGAGACAATGGGGAAA |
|  | ERF039-qRT-R | CGGCTTCTTGAATGTCTTTAGG |
|  | LOX1-qRT-F | CGGAAACAAAATCACTCTTCGT |
|  | LOX1-qRT-R | CGGAAACAAAATCACTCTTCGT |
|  | anac048-qRT-F | TGACTGATGATGGTGTTGATGA |
|  | anac048-qRT-R | TCACCTCTTGAGTTAGTGACAC |
|  | NAC100-qRT-F | ATGAGTACAGGCTTGAAGGAAA |
|  | NAC100-qRT-R | GACCGGTTCTGTTTTGGTTTTA |
|  | NAC079-qRT-F | CATGGGAGTTGCCATATAAAGC |
|  | NAC079-qRT-R | TGCCTCGGAAGATCTCTTTATC |
|  | NAC003-qRT-F | CCGATTGGGTTATACACGAGTA |
|  | NAC003-qRT-R | ACACCAGAAGAAGAAGGAGAAG |
|  | LTP4-qRT-F | AAAAAGTTGAACGGTATGGCTC |
|  | LTP4-qRT-R | GCTAGACTTGGATTAACCCCTT |
|  | IAA7-qRT-F | CTCCGTTGATCTCAAAAACGTT |
|  | IAA7-qRT-R | GTTCTTCCTGTAGTTCCTCACA |
| Subcellular localization | AHL9-Gateway-F | GGGGACAAGTTTGTACAAAAAAGCAGGCTGCATGGATCGAAGAGATGCAATGG |
|  | AHL9-Gateway-R | GGGGACCACTTTGTACAAGAAAGCTGGGTGACCGCGCATTAAATCAATATCAG |
